# SLP-Net: A Dual-Level Contrastive Learning Framework with Stripe Attention for Elongated Pepper Detection in Complex Field Environments

**DOI:** 10.3390/plants15101521

**Published:** 2026-05-15

**Authors:** Jiangquan Zeng, Jiangzhang Zhu, Guoxiong Zhou, Peng Wang

**Affiliations:** 1 College of Computer and Mathematics, Central South University of Forestry and Technology, Changsha 410004, China; zengjiangquan88@gmail.com; 2 College of Electronic Information and Physics, Central South University of Forestry and Technology, Changsha 410004, China; t20252936@csuft.edu.cn

**Keywords:** pepper detection, elongated object detection, contrastive learning

## Abstract

Pepper detection in field images is difficult because the fruits can differ substantially in appearance, and many are partially covered by nearby leaves. Localization becomes less reliable when a pepper is slender or when only part of its contour is visible. SLP-Net was developed for this setting. Rather than increasing model size, it is designed to preserve shape cues that are easily weakened in cluttered field scenes. This makes the detector less sensitive to differences among pepper instances and to cases in which the visible region is incomplete. On PP-Set, SLP-Net outperforms the compared detectors, with clearer gains at higher IoU thresholds and on small targets. A similar pattern is observed on CH-Set, where disease, deformation, and stronger background interference further increase the difficulty of detection. Overall, these results indicate that SLP-Net remains more stable when pepper targets vary more strongly in geometry, surface condition, and visibility.

## 1. Introduction

Pepper is widely cultivated and has practical importance in agricultural production. With the development of digital agriculture and intelligent farm management, automatic pepper detection is being used in tasks such as yield estimation, crop monitoring, and robotic harvesting [1,2,3,4]. Despite these applications, pepper detection in field scenes is still challenging [5,6,7]. Field images often contain substantial variation in illumination, partial leaf occlusion, and clear appearance differences among fruits. Under these conditions, missed detections and inaccurate localization remain common. Such errors can directly affect downstream tasks, including yield estimation and subsequent field management decisions [8]. For this reason, reliable pepper detection under complex field conditions is still an important problem in agricultural vision.

Earlier pepper detection methods mainly depended on handcrafted visual descriptors and manually designed physical cues. Typical examples include color-space transformation combined with threshold segmentation for candidate extraction [9] and edge-based operators for boundary localization [10]. Later studies introduced machine learning methods such as support vector machines (SVM) to improve discrimination based on color and texture features [11]. Other sensing strategies were also investigated, including point cloud clustering [12] and thermal imaging [13]. These methods can perform reasonably well in relatively controlled environments, but they are often less reliable in real field scenes. Changes in illumination can reduce the usefulness of color cues, while dense foliage may hide or break the visible boundary of the fruit. When peppers overlap or are only partially visible, methods that rely mainly on low-level visual information are more likely to miss targets or produce imprecise bounding boxes. This makes their application in field deployment more limited.

Recent pepper detection studies have mostly improved detectors in terms of efficiency and generic feature extraction, rather than directly modeling the structural properties of pepper targets. Most of these methods are still built on single-stage YOLO architectures, with improvements mainly introduced through lightweight convolutions, attention modules, model compression, or stronger multi-scale features to improve accuracy and reduce computational cost [14,15,16,17]. A similar tendency can also be seen in Transformer-based detectors. For example, GP-DETR reduces confusion between peppers and visually similar plants by strengthening the feature pyramid [18]. Existing pepper studies also indicate that small targets are an important source of difficulty in field scenes. Yin et al. [19] compared YOLOv5 with Mask R-CNN and found that YOLOv5 achieved favorable real-time performance while remaining effective for small peppers. This makes small-object detection methods particularly relevant to pepper detection, since they aim to preserve high-resolution spatial cues when pixel coverage is limited. For example, PC-YOLO11s improves small-object localization by adding a P2 detection layer, removing the P5 layer, and enhancing spatial coordinate modeling [20]. Overall, existing studies have improved detector efficiency, multi-scale representation, and small-object localization, but they still focus mainly on optimizing generic detection components. In contrast, the properties that make peppers difficult to detect, particularly elongated contours, pronounced shape variation, and fragmented boundaries under occlusion, are still rarely modeled directly.

**Figure 1:**
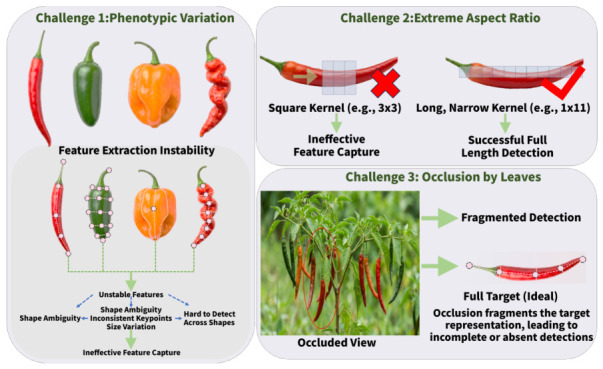
The three main challenges in chili pepper detection.

Field images make pepper detection difficult because the fruit and the surrounding scene can both vary substantially (Figure 1). Pepper itself is not visually consistent. Even within the same category, peppers may be slender, thick, or curved, and their surfaces may change during ripening and senescence, from smooth to wrinkled. As a result, samples that look less typical are harder to handle reliably. Shape is also a challenge. Mature peppers are usually elongated, often with aspect ratios between 5:1 and 10:1. Most standard detectors still rely mainly on square convolutional patterns. For long and thin peppers, this mismatch can make useful boundary cues harder to preserve. The background adds further difficulty. Leaves and branches often cover part of a pepper, so the visible region may be broken into several separated parts even when the fruit itself is intact. In these cases, detection becomes harder because the model no longer sees one complete and clear target region. Peppers under heavy occlusion are therefore more likely to be missed.

Motivated by these challenges, we focus on three factors that repeatedly affect pepper detection in the field, namely large phenotypic variation, elongated object geometry, and frequent leaf occlusion. To address them in one framework, we propose SLP-Net for accurate pepper detection in complex agricultural scenes. The main contributions are as follows:

(1) We introduce a dual-level contrastive learning mechanism, termed ICC, to reduce the effect of morphological variation. ICC acts on both individual samples and category-level features, making feature learning less sensitive to large appearance changes among peppers. As a result, the detector is more reliable across different pepper forms.

(2) We develop a multi-scale stripe-based local spatial attention module, termed MS-LSAM, for elongated targets. Instead of relying only on square convolutional patterns, MS-LSAM uses stripe convolutions at different scales to capture the long, parallel edges that appear frequently in peppers. This makes the extracted features better suited to slender objects and improves box localization.

(3) We design a topology reconstruction loss, termed TR-Loss, to improve robustness under occlusion. TR-Loss randomly masks part of the MS-LSAM stripe feature inside each ground-truth RoI, fills the masked region with the mean response from the visible area, and measures the reconstruction error with an MSE loss. This encourages the model to preserve the overall structure of peppers when parts of the object are occluded.

## 2. Materials and Methods

### 2.1. Dataset

We evaluated SLP-Net on PP-Set [21], a field-collected pepper detection dataset from real cultivation scenes. PP-Set contains 4581 high-resolution images from three categories: Green Pepper, Green Chili, and Red Chili. The dataset is split into 3203 training images, 916 validation images, and 462 test images. In total, it includes 16,955 annotated objects. Table 1 summarizes the instance distribution. The training, validation, and test splits contain 11,359, 3576, and 2020 instances, respectively. At the category level, Green Chili has 7695 instances, Red Chili has 6975, and Green Pepper has 2285. All images are resized to 640×640, and the annotations are converted to Pascal VOC format for bounding-box detection.

PP-Set remains difficult because peppers vary considerably in geometry and appearance. Their aspect ratios span a wide range, and individual fruits may appear nearly straight or clearly curved. Surface appearance also changes across growth stages: some fruits look smooth, whereas others show wrinkles and irregular contours. These variations increase intra-class diversity and make visual cues less stable. Occlusion is also common in field scenes. Peppers are often partially covered by leaves, and overlap among fruits frequently occurs in dense planting areas. As a result, some objects are visible only in fragmented parts, which reduces the cues available for accurate localization.

We also use CH-Set [22] for cross-scenario evaluation. Its test split is used as an independent test set and contains 95 high-resolution images in which healthy and diseased peppers coexist. Some peppers in CH-Set show more extreme aspect ratios, making elongated structures harder to capture completely. Diseased areas also alter the original texture and color of the fruit, which increases recognition difficulty under cross-scene appearance variation. For this reason, CH-Set is used to evaluate robustness under simultaneous structural and appearance changes.

During training, each image is processed by two independently sampled augmentation pipelines to generate the paired views required by ICC. The augmentations include random horizontal flipping, color jittering, and region masking. Because the two pipelines are sampled independently, the resulting views of the same image differ in both color and spatial arrangement. This allows ICC to construct paired samples with appearance variation while preserving object identity for instance-level and class-level contrastive learning. The bounding boxes are transformed consistently with the corresponding augmentations and then normalized to the [0,1] range, so that RoIAlign can extract features from the correct object regions.

### 2.2. SLP-Net Architecture

**Figure 2:**
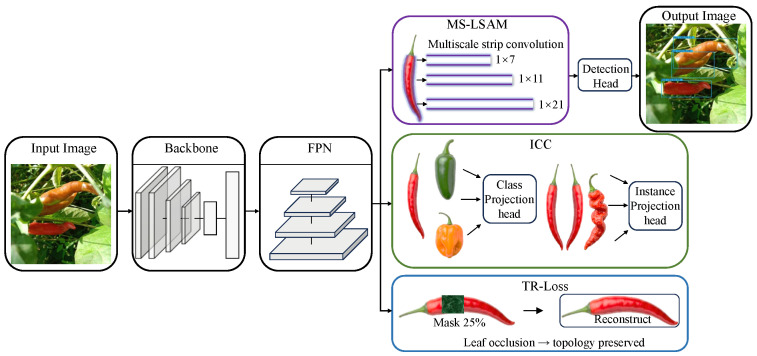
The architecture of SLP-Net.

Figure 2 shows the overall architecture of SLP-Net. Given an input image $I\in R^{3\times 640\times 640}$, ShuffleNetV2 1.0× is adopted as the feature extractor [23] to obtain multi-scale features. After the initial convolution and pooling layers, three feature maps, $\left\{P_{2},P_{3},P_{4}\right\}$, are obtained with spatial sizes of 80×80, 40×40, and 20×20, and channel numbers of 116, 232, and 464, respectively. These feature maps are then fed into GhostPAN [24] to construct a four-level feature pyramid, $\left\{F_{2},F_{3},F_{4},F_{5}\right\}$. The corresponding downsampling factors are 8, 16, 32, and 64, and all pyramid levels are unified to 96 channels. Each pyramid level is subsequently refined by MS-LSAM. This module consists of a channel-attention branch and a spatial-attention branch, whose outputs are combined through element-wise multiplication. In the spatial branch, stripe convolutions with three kernel sizes, 1×7, 1×11, and 1×21, are adopted to capture elongated structures. At each scale, horizontal and vertical responses are computed separately and then merged by maximum-response fusion to generate the final spatial attention map. MS-LSAM produces the refined features $\left\{F_{2}^{\prime },F_{3}^{\prime },F_{4}^{\prime },F_{5}^{\prime }\right\}$, while the stripe features are retained for the reconstruction loss. The refined pyramid features are finally sent to the NanoDetPlusHead [25] for classification and bounding box regression. During training, two auxiliary objectives are further introduced. ICC takes RoI features from $P_{3}$ and $P_{4}$ and maps them into a 128-dimensional embedding space with separate heads for instance-level and class-level contrastive learning. TR-Loss is applied to the stripe features, where 25% random masking is used for feature reconstruction under partial occlusion.

The subsequent sections provide detailed descriptions of each component.

### 2.3. MS-LSAM

**Figure 3:**
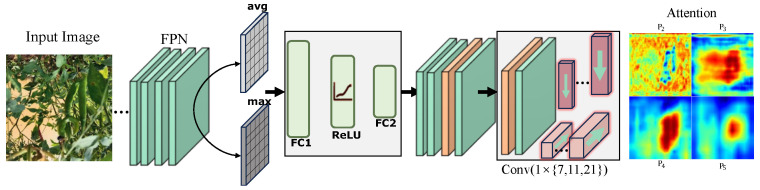
Illustration of MS-LSAM.

Peppers in field scenes usually appear as elongated tubular objects with approximately parallel edges, whereas leaves more often form broader planar regions with less regular internal patterns. Standard convolutions with fixed square kernels are not well suited to this shape difference and tend to mix pepper responses with nearby background textures. Deformable convolution can partially alleviate this problem by adapting the sampling positions, but it also increases optimization difficulty and computational cost [26]. When peppers and leaves share similar colors, unconstrained offsets may drift toward irrelevant background regions. MS-LSAM is designed for this setting, as shown in Figure 3.

Given an input feature map $x\in R^{B\times C\times H\times W}$, MS-LSAM first applies channel attention to reweight informative channels. Global average pooling and global max pooling are performed on *x* to obtain two channel descriptors, which are then fed into a shared multilayer perceptron implemented by two 1×1 convolutional layers with a ReLU activation in between. The channel reduction ratio is set to r=16. The resulting channel attention map is:

(1) $$M_{\mathrm{CA}}\left(x\right)=\sigma \left(\mathrm{MLP}\left(F_{\mathrm{avg}}\right)+\mathrm{MLP}\left(F_{\max}\right)\right),$$

and the feature map is reweighted accordingly:

(2) $$x^{\prime }=x⊙M_{\mathrm{CA}}\left(x\right),$$

where ⊙ denotes element-wise multiplication.

Spatial attention is then computed from $x^{\prime }$. Average pooling and max pooling along the channel dimension produce two spatial descriptors, which are concatenated into a compressed feature map $F_{s}\in R^{B\times 2\times H\times W}$. MS-LSAM uses three strip-convolution scales, with kernel sizes k∈{7,11,21}, to handle peppers with different visible spans in field images. The three scales are introduced because pepper targets in the dataset vary in aspect ratio and visible extent under scale variation, viewpoint change, and partial occlusion. Under these changes, one strip scale is not sufficient for all cases. Using multiple kernel lengths also helps cover both local and extended structure: shorter kernels respond to nearby boundary cues, whereas longer kernels cover more spatially extended slender regions. In practice, 1×7, 1×11, and 1×21 provide short-, medium-, and long-range strip responses, respectively. Their combination provides responses over different spatial ranges, which is more suitable for peppers whose visible extent varies within the same dataset. For each scale, a horizontal strip convolution with kernel size 1×k and a vertical strip convolution with kernel size k×1 are applied to $F_{s}$. Their responses are fused by element-wise maximization:

(3) $$A^{\left(k\right)}=\max\left(A_{h}^{\left(k\right)},A_{v}^{\left(k\right)}\right),$$

where $A^{\left(k\right)}\in R^{B\times 1\times H\times W}$ is the strip response at scale *k*. The three strip responses are then averaged to obtain the fused spatial response, and the spatial attention map is generated by applying a sigmoid activation to it. The final output feature map is written as:

(4) $$x^{\prime \prime }=x^{\prime }⊙M_{\mathrm{SA}}\left(x^{\prime }\right),$$

Besides the enhanced feature map $x^{\prime \prime }$ used by the detection head, MS-LSAM also preserves the three scale-specific strip responses as intermediate structural features. They are concatenated along the channel dimension to form $S\in R^{B\times 3\times H\times W}$, which is further used by TR-Loss under partial occlusion.

### 2.4. ICC

Peppers vary considerably across cultivars and growth stages, and such variation often destabilizes detection. Conventional detection losses supervise classification and box regression, but they do not explicitly shape the feature distribution within a category. As a result, peppers with the same class but markedly different shapes may still remain separated in the feature space. A slender pepper and a short, thick one, for instance, can elicit quite different visual responses. Without extra feature-level guidance, features from the same category may become overly scattered, which in turn weakens generalization to peppers with unseen morphologies.

Bringing same-class samples closer is therefore useful in this setting. This idea is related to supervised contrastive learning, although most existing formulations are designed for image classification rather than object detection. The gap is not only in the task objective. A detector must classify objects and localize them at the same time, and the features supporting these two objectives are not always aligned. The sample structure also differs: classification typically assigns one label to one image, whereas detection must handle multiple objects and multiple boxes within a single image. As a consequence, positive and negative pairs are less straightforward to construct. In addition, most contrastive methods operate on global image representations, while detection depends on features tied to specific object regions.

**Figure 4:**
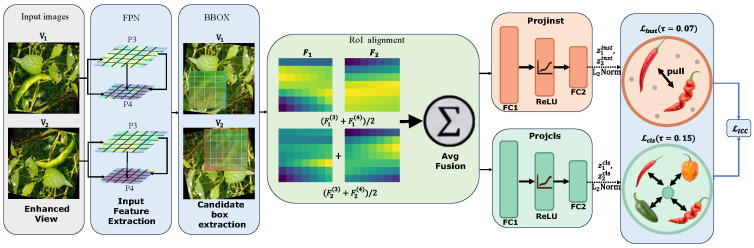
A schematic diagram of an ICC.

ICC (Figure 4) is introduced for this reason. It injects contrastive supervision directly at the object level through two branches. One branch keeps the same region consistent across augmented views. The other reduces variation among peppers from the same category. Given two augmented inputs $V_{1}$ and $V_{2}$, RoI features are extracted from the two branches after the backbone and FPN. RoI Align is then applied on the intermediate pyramid levels $P_{3}$ and $P_{4}$, producing fixed-size region features $F_{1}^{\left(3\right)},F_{1}^{\left(4\right)}$ and $F_{2}^{\left(3\right)},F_{2}^{\left(4\right)}$. The features from $P_{3}$ and $P_{4}$ are averaged as follows:

(5) $$F_{1}=\frac{F_{1}^{\left(3\right)}+F_{1}^{\left(4\right)}}{2},\;F_{2}=\frac{F_{2}^{\left(3\right)}+F_{2}^{\left(4\right)}}{2},$$

where $F_{1}$ and $F_{2}$ denote the fused region representations from the two augmented views. The choice of $P_{3}$ and $P_{4}$ is empirical. These two levels retain sufficient semantic content for category discrimination while preserving the spatial detail needed for region-wise comparison.

Each RoI feature is sent to two projection heads, one for instance-level contrast and the other for class-level contrast. For the paired region features $F_{1}$ and $F_{2}$ from views $V_{1}$ and $V_{2}$, the embeddings are defined as follows:

(6) $$\begin{array}{ccc} z_{1}^{\mathrm{inst}} & ={\mathrm{Proj}}_{\mathrm{inst}}\left(F_{1}\right),\;z_{1}^{\mathrm{cls}} & ={\mathrm{Proj}}_{\mathrm{cls}}\left(F_{1}\right),\\ z_{2}^{\mathrm{inst}} & ={\mathrm{Proj}}_{\mathrm{inst}}\left(F_{2}\right),\;z_{2}^{\mathrm{cls}} & ={\mathrm{Proj}}_{\mathrm{cls}}\left(F_{2}\right), \end{array}$$

where ${\mathrm{Proj}}_{\mathrm{inst}}\left(\cdot \right)$ and ${\mathrm{Proj}}_{\mathrm{cls}}\left(\cdot \right)$ denote the instance-level and class-level projection heads, respectively. The two heads share the same architecture, consisting of a fully connected layer ${\mathrm{FC}}_{1}\in R^{96\times 256}$, a ReLU activation, a second fully connected layer ${\mathrm{FC}}_{2}\in R^{256\times 128}$, and an $\ell_{2}$ normalization layer that outputs a 128-dimensional embedding.

The four embeddings are optimized with two supervised contrastive objectives. For a paired RoI, $z_{1}^{\mathrm{inst}}$ and $z_{2}^{\mathrm{inst}}$ are treated as a positive pair, so that the same pepper remains close across the two augmented views. The class-level branch uses category labels instead. Peppers from the same class are pulled toward a more compact distribution even when their lengths, thicknesses, or curvatures differ substantially. Both branches use the supervised contrastive loss:

(7) $$L_{\mathrm{SupCon}}=-\log\frac{\sum_{y_{i}=y_{j}}\exp\left(z_{i}\cdot z_{j}/\tau \right)}{\sum_{k=1}^{N}\exp\left(z_{i}\cdot z_{k}/\tau \right)},$$

where $z_{i}\cdot z_{j}$ denotes the cosine similarity between normalized embeddings, and τ is the temperature parameter.

The two branches use different temperatures because they enforce different degrees of compactness in the embedding space. In the instance-level branch, the two views come from the same pepper, so their features should be pulled closer. We therefore use a smaller temperature, $\tau_{\mathrm{inst}}=0.07$, to make positive-pair matching more selective. The class-level branch is looser. Even within the same category, peppers can still differ in length, thickness, curvature, and visible extent, so compressing same-class samples too strongly would discard useful geometric variation. We therefore set a larger temperature, $\tau_{\mathrm{cls}}=0.15$, so that samples from the same class remain clustered without being forced into an overly tight distribution. Both temperatures were chosen empirically according to these branch-specific roles and kept fixed in all experiments. The overall ICC loss is defined as

(8) $$L_{\mathrm{ICC}}=w_{\mathrm{inst}}L_{\mathrm{inst}}+w_{\mathrm{cls}}L_{\mathrm{cls}},$$

where $w_{\mathrm{inst}}=0.05$ and $w_{\mathrm{cls}}=0.3$. We keep the instance-level term light because it mainly serves as an auxiliary constraint for aligning two views of the same RoI, rather than acting as a primary training signal. We assign a larger weight to the class-level term because the same pepper category still contains substantial variation in shape and appearance, making control of intra-class spread more necessary in this branch. Under these weights, the contrastive losses regularize the feature space while remaining subordinate to the main detection objective.

### 2.5. TR-Loss

Peppers in field scenes are often occluded by leaves or neighboring fruits, which leads to incomplete object responses and confusion with surrounding leaf textures. This issue becomes more pronounced for elongated objects, because only part of a pepper may be visible even though the detector still needs to localize the whole instance.

We apply TR-Loss to the strip responses of MS-LSAM to improve robustness to such partial occlusion. Denote the strip-response map by $S\in R^{B\times 3\times H\times W}$, where the three channels correspond to the three strip-kernel branches and H,W are the spatial dimensions of the selected FPN level. These responses are used in place of generic backbone features because they preserve directional structure more explicitly and retain shape continuity across multiple receptive fields.

For each ground-truth box $b=[x_{1},y_{1},x_{2},y_{2}]$, the coordinates are projected onto the assigned FPN level:

(9) $$b^{\prime }=\left[x_{1}^{\prime },y_{1}^{\prime },x_{2}^{\prime },y_{2}^{\prime }\right]=\left[x_{1}/\mathrm{stride},\;y_{1}/\mathrm{stride},\;x_{2}/\mathrm{stride},\;y_{2}/\mathrm{stride}\right],$$

where stride is the downsampling factor of that level. Each box is assigned to the FPN level with the closest stride-to-scale match, and the mapped box $b^{\prime }$ defines an RoI on the strip-response map, from which we crop $S_{\mathrm{RoI}}\in R^{3\times h\times w}$. To avoid degenerate regions, the minimum feature-space box size is set to 4 pixels. For elongated objects, it is sufficient that either the height or width satisfies this threshold.

A rectangular binary mask $M\in R^{1\times h\times w}$ is generated for each RoI and shared across all three channels. The mask size is set to 25% of the cropped height and width, with a minimum of 2 pixels along each dimension, and its position is uniformly sampled within the RoI. The masked response is obtained by element-wise multiplication between $S_{\mathrm{RoI}}$ and *M*. The masked region is then filled with the channel-wise mean computed from the visible locations, yielding a reconstructed response $S_{\mathrm{recon}}$. This step does not aim to reconstruct the missing region accurately. It instead perturbs the local strip response while keeping the global channel statistics of the RoI unchanged.

For the *n*-th positive RoI, TR-Loss is defined as the mean squared error between the reconstructed response and the original cropped response:

(10) $$L_{\mathrm{TR}}^{\left(n\right)}=\frac{1}{3hw}\sum_{c=1}^{3}\sum_{i=1}^{h}\sum_{j=1}^{w}{\left(S_{\mathrm{recon},c,i,j}^{\left(n\right)}-S_{\mathrm{RoI},c,i,j}^{\left(n\right)}\right)}^{2},$$

We average this term over all positive RoIs in the current batch to obtain the final TR-Loss. When no positive RoI is present, we set $L_{\mathrm{TR}}=0$. TR-Loss is used only during training and is removed at inference.

The overall loss function is

(11) $$L_{\mathrm{total}}=L_{\mathrm{cls}}+L_{\mathrm{loc}}+w_{\mathrm{ICC}}L_{\mathrm{ICC}}+w_{\mathrm{TR}}L_{\mathrm{TR}},$$

where $w_{\mathrm{ICC}}=0.35$ and $w_{\mathrm{TR}}=0.1$. We use a moderate weight for $L_{\mathrm{ICC}}$ because it is applied on multi-scale FPN features and serves as an auxiliary constraint throughout the feature hierarchy. We keep $w_{\mathrm{TR}}$ smaller because $L_{\mathrm{TR}}$ is applied only to positive RoIs and mainly regularizes local strip responses. Based on experience, larger values made training less stable, while $w_{\mathrm{TR}}=0.1$ gave the best validation results.

## 3. Results

### 3.1. Experimental Environment and Training Details

All experiments were conducted on the same workstation, equipped with an AMD EPYC 9754 128-core CPU and an NVIDIA GeForce RTX 4090 D GPU with 24 GB memory. The software environment was built on Ubuntu 18.04 with CUDA 11.4 and cuDNN V7.4.2. All models were implemented in Python 3.9 using PyTorch-GPU 1.12.1. To ensure a fair comparison, all competing methods were trained and evaluated on the same pepper dataset using the same train/validation split, the same input resolution of 640×640, and the same evaluation protocol.

All compared models were trained for 100 epochs with a batch size of 8. The optimizer was SGD, with momentum and weight decay set to 0.937 and 0.0005, respectively. The initial learning rate was set to $1\times 10^{-5}$ and updated using a cosine annealing schedule, with the minimum learning rate ratio set to 0.01. No dataset-specific hyperparameter tuning beyond the above common settings was performed for the compared methods. For model initialization, publicly available official pretrained weights were adopted whenever available. Specifically, the YOLO-series models were initialized with official pretrained weights pretrained on COCO, CSPNet was initialized with its official pretrained weights pretrained on ImageNet, and ShuffleNetV2, GhostNet, and MobileNet were initialized with their respective official pretrained weights pretrained on ImageNet.

### 3.2. Evaluation Metrics

We use AP and mAP as the main evaluation metrics. AP at a given IoU threshold is defined as

(12) $$AP=\int_{0}^{1}P\left(r\right)\;dr,$$

where P(r) denotes the precision–recall curve. mAP is the average AP over all object categories:

(13) $$mAP=\frac{1}{N}\sum_{i=1}^{N}AP_{i},$$

where *N* is the number of categories.

We further report AP@50 and AP@75 at IoU thresholds of 0.5 and 0.75, respectively, so as to compare performance under different localization strictness.

Following the COCO protocol, AP@S, AP@M, and AP@L are also reported for small, medium, and large objects. These three groups are defined by object area below 32×32, between 32×32 and 96×96, and above 96×96 pixels, respectively, and are used to assess detector performance across different object scales.

We use one metric, Edge Concentration Score (ECS), to examine whether the spatial attention map assigns relatively stronger responses near proxy boundary regions. The proxy region is defined as a 3-pixel-wide strip along each side of the ground-truth bounding box. Before computing the metric, the ground-truth box is mapped to the spatial resolution of the attention map. ECS is defined as the ratio between the mean attention intensity on this bounding-box edge strip and the image-wide mean attention intensity over the full map:

(14) $$\mathrm{ECS}=\frac{\mu_{E}}{\mu_{G}}=\frac{\frac{1}{\left|E\right|}\sum_{p\in E}a_{p}}{\frac{1}{H\times W}\sum_{i,j}a_{ij}}.$$

Here, $a_{p}$ denotes the attention intensity at pixel *p*, *E* denotes the set of pixels in the bounding-box edge strip, and H×W denotes the spatial size of the attention map. An ECS value greater than 1 means that the mean attention intensity on the proxy boundary strip is higher than the image-wide average, whereas a value below 1 means that it is lower than the image-wide average.

### 3.3. Ablation Study

Table 2 lists the ablation results of ICC, MS-LSAM, and TR-Loss under the same data split and training setting. Among the three modules, MS-LSAM contributes the largest gain. After it is added to the baseline, mAP increases from 40.8% to 52.5%, and AP@75 as well as AP@S also improve clearly.

ICC brings a smaller overall gain than MS-LSAM, but the improvement on AP@75 is more noticeable. Its effect is also relatively stable on medium-sized objects, indicating that ICC is more helpful for feature discrimination and localization quality than for broad gains on all metrics.

TR-Loss alone improves the baseline only slightly. Its effect becomes more visible when it is used together with ICC or MS-LSAM. In this setting, TR-Loss behaves more like a supplementary constraint than a major source of improvement. Across the ablation results, two-module variants consistently outperform the corresponding single-module counterparts, and the full SLP-Net achieves the best overall performance.

### 3.4. Effectiveness of ICC Module

We compare ICC with several representative contrastive learning methods, including SimCLR, BYOL, MoCo-v3, and SupCon. The quantitative results are reported in Table 3. Among all compared methods, ICC gives the best results on every reported metric, reaching 54.6% mAP and 59.8% AP@75. It also achieves the highest AP on small, medium, and large objects. By contrast, SimCLR, BYOL, and MoCo-v3 all remain below the Baseline, while SupCon is the strongest among the comparison methods but still falls short of ICC. This comparison suggests that directly introducing a generic contrastive objective is insufficient for this task, whereas the dual-level design of ICC is better suited to pepper detection.

A closer look at the compared methods reveals a clear pattern. SimCLR shows the largest drop relative to the Baseline. BYOL and MoCo-v3 narrow this gap, but their improvements are still limited, especially on AP@75. SupCon performs much better once label supervision is introduced, which makes it clearly stronger than the unsupervised methods. Even so, ICC remains consistently better than SupCon across all reported metrics. These results indicate that, for this task, contrastive learning benefits from modeling both instance-level consistency and class-level compactness rather than relying on a single generic objective.

### 3.5. Effectiveness of MS-LSAM Module

Table 4 compares MS-LSAM with several widely used attention mechanisms. MS-LSAM achieves the highest mAP, AP@50, and AP@75, reaching 54.6%, 93.6%, and 59.8%, respectively. It also gives the best AP@S and AP@M, with values of 36.2% and 41.1%. Relative to the other attention variants, the gains are larger on AP@75 and AP@S than on mAP.

Small objects show the largest difference in this comparison. The baseline setting without an additional attention module reaches 12.8% AP@S, and the gains from ECA, SE, GAM, SimAM, and CBAM remain modest compared with MS-LSAM. With MS-LSAM, AP@S rises to 36.2%. This result is consistent with the view that the proposed spatial branch is more useful when the targets are small and harder to localize.

A plausible explanation is related to how elongated peppers appear after repeated downsampling. In deeper feature maps, they often occupy narrow regions and are more easily mixed with nearby leaves or background clutter. Under such conditions, attention modules relying mainly on global pooling or square spatial aggregation may have difficulty highlighting the target region precisely. MS-LSAM instead introduces multi-scale strip convolutions in the spatial branch, which makes the attention map more responsive to elongated layouts. This tendency is most visible in AP@S, while the gains on AP@M and AP@L remain consistent.

**Figure 5:**
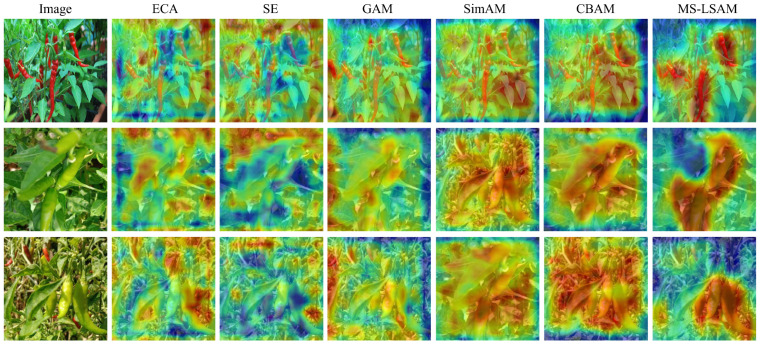
Attention visualization results of different methods. The color intensity indicates the magnitude of attention response, where red represents higher attention values and blue represents lower attention values.

The visualization results in Figure 5 follow the same pattern. In several comparison methods, the highlighted regions are more scattered and often extend beyond the target, especially when the object is small or partially occluded. By contrast, MS-LSAM places stronger responses along the pepper body and its two long boundaries, while suppressing irrelevant background more effectively. This visual behavior is consistent with its higher AP@S.

Table 5 lists ECS values for the aspect-ratio groups. For the baseline methods without a spatial attention module, ECS stays near 1.0 in all four groups. CBAM raises ECS slightly, but the change remains limited across the groups. MS-LSAM shows a larger increase, and the gap from CBAM is more evident in the higher aspect-ratio groups. This tendency agrees with a stronger response around the annotated boundary region when the target shape is more elongated.

CBAM is an appropriate reference here because both modules share the same channel-attention backbone, and their main difference is in the spatial branch: CBAM uses square convolutions, whereas MS-LSAM uses multi-scale strip convolutions. Under this setting, CBAM stays relatively stable across the four groups, while MS-LSAM remains higher throughout. The difference is small in G1 and becomes easier to observe as aspect ratio increases. Read together with the bucketed mAP results in Table 6, this tendency favors MS-LSAM in the groups with larger aspect ratios.

Table 6 contains two related trends. First, the performance of the 3×3 kernel declines markedly as aspect ratio increases. This change suggests that the square kernel is less well suited to targets with more elongated shapes. Second, within the single-strip settings, the better result shifts toward longer strip sizes in the higher aspect-ratio groups. The shortest strip performs best in the low aspect-ratio group, whereas longer strips perform better in the groups containing more elongated targets. These results indicate that different strip lengths are better aligned with different aspect-ratio ranges.

The multi-scale setting shows a clearer advantage in the higher aspect-ratio groups. In the low aspect-ratio group, its gain over the best single-strip setting is limited, whereas the difference is more noticeable in the higher groups, especially in G4. This trend suggests that combining multiple strip scales is more effective than relying on a single strip scale as target shape becomes more elongated. A similar tendency appears in the ECS results, where the advantage of MS-LSAM over CBAM is easier to observe in the middle and higher aspect-ratio groups. Taken as a whole, these results favor the strip-based spatial design in samples with larger aspect ratios.

### 3.6. Effectiveness of TR-Loss

TR-Loss brings a consistent improvement under occlusion. As shown in Table 7, compared with several reconstruction losses used in SLP-Net, it improves overall mAP by 1.3 percentage points. The clearest gains appear on AP@S and AP@75, which increase by 3.6% and 7.3%, respectively. The improvement is therefore more visible on small targets and when localization is evaluated more strictly.

Occlusion often interrupts the visible body of an elongated target, whereas common reconstruction losses still act mainly on pointwise feature differences. MSE and L1 can reduce local reconstruction error, but they do not specify how disconnected visible parts should relate to the whole object. For peppers, this becomes more important when only part of the body can be seen.

TR-Loss works better in this case because random masking prevents the model from relying only on nearby visible responses. Instead, the model has to use incomplete evidence to recover the missing part. As a result, reconstruction is influenced not just by local values, but also by whether the object keeps a plausible overall shape after occlusion. This is also where the larger gains on AP@S and AP@75 become understandable, since small targets and blurred boundaries are more easily affected by missing regions.

The same tendency appears when SSIM Loss and Perceptual Loss are used for comparison. They help preserve structural similarity, but mostly around the visible region itself. TR-Loss adds synthetic occlusion during training, so interrupted target structures appear repeatedly in the learning process. This makes the detector better at handling missing regions at test time, especially for small or partially covered peppers.

### 3.7. Comparison with Other Detection Methods

Table 8 compares SLP-Net with the evaluated detectors. SLP-Net attains the highest mAP, AP@50, and AP@75, reaching 54.6%, 93.6%, and 59.8%, respectively. It also achieves the highest AP@S and AP@M, with values of 36.2% and 41.1%. The gain is therefore visible in AP@75 as well as in the small- and medium-object metrics.

SLP-Net uses 4.2 M parameters and 2.43 G FLOPs, both lower than those of CSPNet, while achieving higher mAP and AP@75. Relative to lightweight YOLO variants such as YOLOv5n, YOLOv8n, and YOLOX-Nano, SLP-Net uses more parameters, but the differences are larger on AP@75, AP@S, and AP@M. Its inference speed is 175 FPS. Although this is lower than that of the fastest lightweight baselines, it remains within the real-time range. These comparisons suggest that the accuracy gain is not accompanied by a large increase in computational cost.

Lightweight methods are a useful reference on this dataset. GhostNetV2 reaches 53.8% mAP, which is close to SLP-Net in mAP, but SLP-Net remains higher on AP@75, AP@S, AP@M, and AP@L. The gap is larger for YOLOv5n, YOLOv8n, and YOLOX-Nano, whose scores are lower on most reported metrics. Although some of these models are smaller or faster, their AP@75, AP@S, and AP@M values remain below those of SLP-Net.

**Figure 6:**
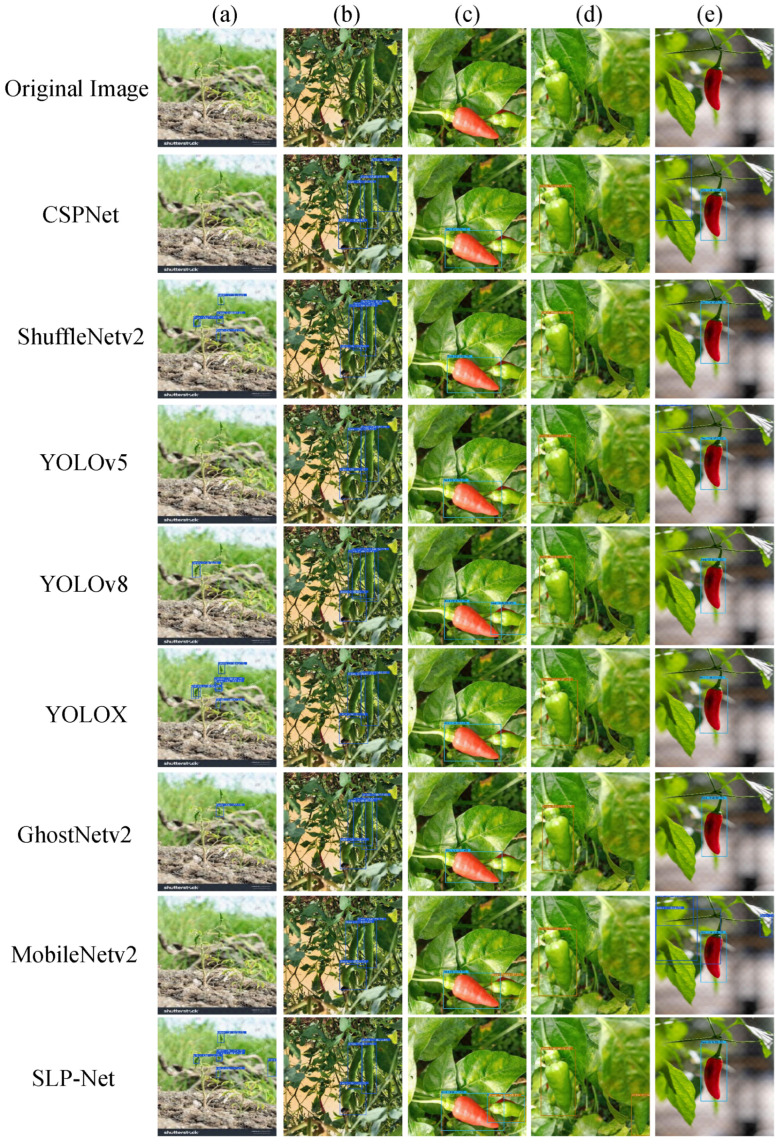
Performance comparison visualization of different detection methods.

Figure 6 shows visual examples corresponding to the quantitative comparison. In Figure 6a, the targets are small and the image is affected by low resolution and strong background interference. Under this condition, SLP-Net localizes all visible pepper instances, whereas CSPNet, YOLOv5n, and MobileNetV2 miss some of them. In Figure 6b, red peppers are heavily occluded by green peppers. SLP-Net still detects the occluded instances, while the compared models shown in the figure miss them. These examples are in line with the larger metric gaps observed on small targets and under stricter localization settings.

## 4. Discussion

CH-Set is more challenging than PP-Set because it contains diseased peppers, more extreme aspect ratios, and heavier background interference. We therefore used it as an independent test of whether SLP-Net remains effective under this cross-scene setting.

Table 9 shows that SLP-Net still performs competitively on this dataset. Its mAP drops from 54.6% on the original test set to 48.2%, but it remains ahead of the compared methods on CH-Set. The advantage is still clear on AP@50, which reaches 84.5%. SLP-Net also keeps relatively strong results on small and medium-sized objects, with AP@S of 27.8% and AP@M of 34.2%. In other words, the gain of SLP-Net is still visible after the test scene changes.

The degradation is more obvious for several comparison models. GhostNetV2, for example, decreases from 53.8% mAP to 45.5%, a drop of 8.3 percentage points. This difference becomes more noticeable when target appearance changes more sharply, such as in diseased samples or more elongated peppers. Under these conditions, models that rely more heavily on appearance cues tend to become less stable.

**Figure 7:**
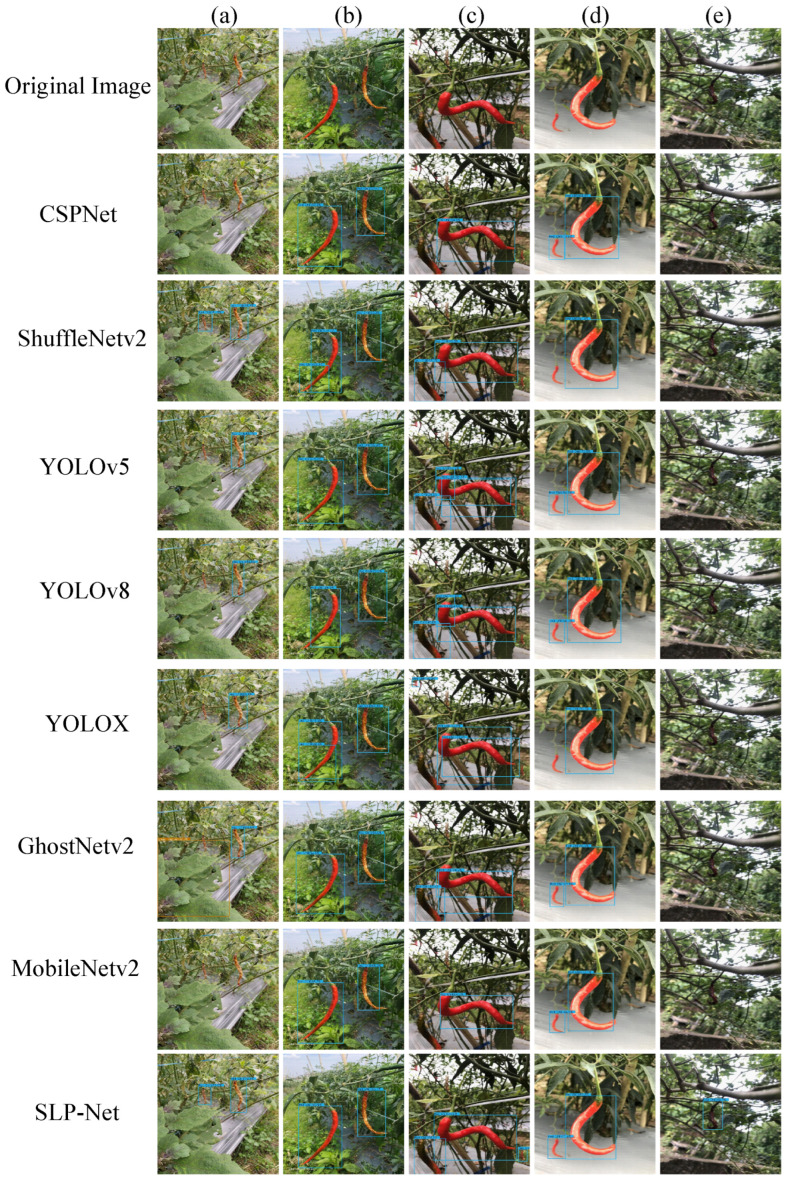
Visualization of generalization performance of different detection methods on the CH-Set dataset.

The visual examples in Figure 7 show the same pattern. In Figure 7a,e, SLP-Net detects diseased peppers that do not appear in PP-Set, whereas most comparison models fail on these atypical samples. In Figure 7b,c, several models produce fragmented boxes or incomplete detections for extremely elongated targets, while SLP-Net still gives a more continuous prediction. The gap is also visible in small or partially occluded cases, where the other detectors miss targets more often. Taken together, the cross-dataset results show that SLP-Net is less sensitive to changes in pepper appearance and field conditions than the compared detectors.

## 5. Conclusions

SLP-Net is proposed for field pepper detection, where lightweight models often struggle with large phenotypic variation, elongated fruit shapes, and frequent leaf occlusion. Its design follows the visual characteristics of peppers in field scenes instead of relying on a larger model size. In these images, peppers are often long and thin, while standard detectors still depend mainly on square receptive patterns. Occlusion can further leave only part of the target visible. Under such conditions, structural information is more easily weakened during feature extraction. To address this issue, SLP-Net is introduced to improve feature stability across instances, retain clearer boundary information for elongated targets, and remain more robust when the visible region is incomplete.

Model size alone does not explain the difference here. A more relevant issue is whether the detector can preserve useful shape cues when the target becomes less regular. This is consistent with the cross-dataset results, where SLP-Net changes less under diseased samples, larger aspect-ratio variation, and stronger background clutter. For pepper detection, this appears to be more useful than simply increasing network depth or model scale.

The current design also has clear limitations. The strip-based spatial module is better matched to elongated targets, but it is less suitable for objects with a strong curvature or an articulated structure. The contrastive branch is defined on RoI features, so relations among nearby objects in crowded scenes are not modeled directly. The occlusion setting used in TR-Loss is also simplified, because random masking can only serve as a rough approximation of occlusion in real field images.

There is still room to improve several parts of the framework. Targets with stronger curvature may need a more flexible geometric operator. The contrastive objective could also be extended to include structural relations among neighboring instances. In addition, random masking could be replaced by occlusion priors estimated from real shape statistics or field scene patterns. Taken together, the current results suggest that geometry-aware feature modeling is helpful for making pepper detection more stable in complex field conditions.

## Figures and Tables

**Table 1:** Instance-level statistics of the PP-Set dataset.

Category	Train	Valid	Test	Total
Green Pepper	1498	512	275	2285
Green Chilli	5342	1488	865	7695
Red Chili	4519	1576	880	6975
Total	11,359	3576	2020	16,955

**Table 2:** Ablation study on pepper dataset. Baseline refers to ShuffleNetV2 1.0× + GhostPAN + NanoDetPlusHead.

Method	mAP	AP@50	AP@75	AP@S	AP@M	AP@L
Baseline	40.8%	87.0%	29.5%	0.0%	24.6%	43.8%
Baseline+ICC	43.0%	88.2%	33.8%	3.8%	26.8%	45.9%
Baseline+MS-LSAM	52.5%	91.2%	51.8%	22.4%	38.5%	54.9%
Baseline+TR-Loss	41.7%	87.3%	30.4%	0.6%	25.4%	44.5%
Baseline+ICC+MS-LSAM	53.3%	92.1%	52.5%	32.6%	39.5%	55.8%
Baseline+ICC+TR-Loss	46.3%	89.5%	40.2%	12.8%	29.7%	48.3%
Baseline+MS-LSAM+TR-Loss	53.1%	91.5%	58.3%	34.1%	38.9%	56.3%
SLP-Net	54.6%	93.6%	59.8%	36.2%	41.1%	58.2%

**Table 3:** Performance comparison of different contrastive learning methods. “–” indicates the baseline model composed of Backbone, MS-LSAM, and TR-Loss, without using any contrastive learning method.

Method	mAP	AP@50	AP@75	AP@S	AP@M	AP@L
–	53.1%	91.5%	58.3%	34.1%	38.9%	56.3%
SimCLR [27]	50.3%	90.2%	54.9%	33.8%	36.5%	53.9%
BYOL [28]	50.9%	91.0%	56.3%	33.2%	37.7%	55.8%
MoCo-v3 [29]	52.5%	90.9%	57.4%	33.5%	38.2%	55.7%
SupCon [30]	54.2%	92.4%	59.3%	35.6%	40.4%	57.5%
ICC	54.6%	93.6%	59.8%	36.2%	41.1%	58.2%

**Table 4:** Performance comparison of different attention mechanisms. “–” indicates the baseline model composed of Backbone, ICC, and TR-Loss, without using any attention mechanism.

Method	mAP	AP@50	AP@75	AP@S	AP@M	AP@L
–	46.3%	89.5%	40.2%	12.8%	29.7%	48.3%
ECA [31]	46.9%	90.2%	41.6%	13.4%	30.5%	48.9%
SE [32]	47.2%	89.9%	42.3%	14.1%	30.8%	49.5%
GAM [33]	48.4%	90.7%	43.8%	15.9%	32.4%	50.6%
SimAM [34]	51.1%	91.3%	50.4%	20.5%	34.1%	53.4%
CBAM [35]	52.5%	92.1%	53.9%	26.3%	36.4%	55.7%
MS-LSAM	54.6%	93.6%	59.8%	36.2%	41.1%	58.2%

**Table 5:** Edge concentration analysis by object aspect-ratio bucket. G1–G4 correspond to AR ranges <2.5, 2.5≤AR<5, 5≤AR<8, and ≥8, respectively. All models use the same backbone (Backbone+ICC+TR-Loss).

Method	G1	G2	G3	G4
ECA [31]	1.02	1.02	1.00	0.95
SE [32]	1.04	1.05	1.08	1.13
GAM [33]	0.92	0.89	0.93	1.03
SimAM [34]	1.05	1.02	1.04	1.02
CBAM [35]	1.05	1.04	1.06	1.07
MS-LSAM	1.22	1.30	1.27	1.28

**Table 6:** mAP by object aspect-ratio bucket for each kernel scale. G1–G4 correspond to AR ranges <2.5, 2.5≤AR<5, 5≤AR<8, and ≥8, respectively. All models use the same backbone (Backbone+ICC+TR-Loss).

Kernel	G1	G2	G3	G4
3×3	54.1	36.4	20.6	10.8
1×7	55.3	39.6	24.0	18.3
1×11	54.6	40.4	25.9	20.5
1×21	54.3	41.3	26.4	21.4
1×7+1×11+1×21	55.8	42.1	26.9	23.5

**Table 7:** Performance comparison of different reconstruction loss functions. “–” indicates the baseline model composed of Backbone, MS-LSAM, and ICC, without using any reconstruction loss function.

Method	mAP	AP@50	AP@75	AP@S	AP@M	AP@L
–	53.3%	92.1%	52.5%	32.6%	39.5%	55.8%
MSE Loss [36]	53.5%	91.9%	52.8%	33.3%	39.7%	55.9%
L1 Loss [37]	53.4%	92.2%	53.6%	32.5%	39.4%	56.1%
SSIM Loss [38]	53.7%	92.0%	54.1%	34.9%	39.8%	57.0%
Perceptual Loss [39]	53.9%	93.4%	56.4%	34.1%	40.2%	57.3%
TR-Loss	54.6%	93.6%	59.8%	36.2%	41.1%	58.2%

**Table 8:** Illustrative performance comparison of different detection methods at 640 × 640 input. ShuffleNetV2 refers to ShuffleNetV2 1.0× + BiFPN + GFLHead.

Method	Params (M)	FLOPs (G)	FPS	mAP	AP@50	AP@75	AP@S	AP@M	AP@L
CSPNet (CSPDarknet-53) [40]	65.2	140	37	41.6%	87.6%	30.8%	28.8%	25.4%	44.4%
ShuffleNetV2 (ShuffleNetV2 1.0×) [23]	2.3	2.01	228	53.3%	90.7%	54.8%	30.5%	35.3%	55.7%
YOLOv5n (CSPDarknet) [41]	1.9	4.5	191	42.4%	88.9%	32.2%	24.6%	23.7%	44.1%
YOLOv8n (C2f + SPPF Backbone) [42]	3.2	8.7	173	42.5%	89.6%	31.5%	27.1%	25.4%	45.2%
YOLOX-Nano (CSPDarknet-Nano) [43]	0.91	1.08	247	45.4%	90.1%	30.5%	30.0%	27.2%	44.9%
GhostNetV2 (GhostNetV2 1.0×) [24]	6.12	2.13	187	53.8%	91.2%	58.7%	35.4%	34.1%	56.3%
MobileNetV2 (MobileNetV2 1.0×) [44]	3.47	1.32	214	36.2%	81.5%	23.4%	25.9%	12.7%	39.4%
SLP-Net (ShuffleNetV2 1.0×)	4.2	2.43	175	54.6%	93.6%	59.8%	36.2%	41.1%	58.2%

**Table 9:** Performance comparison of different detection methods on the CH-Set dataset.

Method	mAP	AP@50	AP@75	AP@S	AP@M	AP@L
CSPNet (CSPDarknet-53) [40]	34.2%	76.5%	24.8%	20.1%	18.3%	37.2%
ShuffleNetV2 (ShuffleNetV2 1.0×) [23]	46.8%	82.3%	46.2%	24.5%	28.6%	48.3%
YOLOv5n (CSPDarknet) [41]	35.6%	78.2%	26.5%	18.3%	19.8%	38.2%
YOLOv8n (C2f + SPPF Backbone) [42]	36.2%	79.1%	27.3%	21.6%	20.1%	38.6%
YOLOX-Nano (CSPDarknet-Nano) [43]	36.8%	79.6%	26.8%	22.5%	21.5%	39.1%
GhostNetV2 (GhostNetV2 1.0×) [24]	45.5%	81.2%	49.8%	25.6%	28.8%	48.6%
MobileNetV2 (MobileNetV2 1.0×) [44]	28.4%	68.9%	16.2%	17.8%	9.2%	31.2%
SLP-Net (ShuffleNetV2 1.0×)	48.2%	84.5%	51.6%	27.8%	34.2%	50.3%

## Data Availability

The original contributions presented in this study are included in the article. Further inquiries can be directed to the corresponding author.
